# No evidence for point mutations in the novel renal cystine transporter AGT1/SLC7A13 contributing to the etiology of cystinuria

**DOI:** 10.1186/s12882-018-1080-5

**Published:** 2018-10-20

**Authors:** Kathrin Olschok, Udo Vester, Sven Lahme, Ingo Kurth, Thomas Eggermann

**Affiliations:** 1 Institute of Human Genetics, University Hospital, Technical University RWTH Aachen, Pauwelsstr. 30, D-52074 Aachen, Germany; 2 Pediatric Hospital, University Hospital, University of Essen, Hufelandstr. 55, 45122 Essen, Germany; 3 Department of Urology, St. Trudpert Hospital, Wilferdinger Str. 67, 75179 Pforzheim, Germany

**Keywords:** Cystinuria, Mutation, AGT1/SLC7A13

## Abstract

**Background:**

Cystinuria is caused by the defective renal reabsorption of cystine and dibasic amino acids, and results in cystine stone formation. So far, mutations in two genes have been identified as causative. The *SLC3A1*/*rBAT* gene encodes the heavy subunit of the heterodimeric rBAT-b^0,+^AT transporter, whereas the light chain is encoded by the *SLC7A9*/ *b*^*0,+*^*AT* gene. In nearly 85% of patients mutations in both genes are detectable, but a significant number of patients currently remains without a molecular diagnosis. Thus, the existence of a further cystinuria gene had been suggested, and the recently identified AGT1/SLC7A13 represents the long-postulated partner of rBAT and third cystinuria candidate gene.

**Methods:**

We screened a cohort of 17 cystinuria patients for *SLC7A13* variants which were negative for *SLC3A1* and *SLC7A9* mutations.

**Results:**

Despite strong evidences for an involvement of *SLC7A13* mutations in cystinuria, we could not confirm a relevant role of *SLC7A13* for the disease.

**Conclusion:**

With the exclusion of *SLC7A13*/AGT1 as the third cystinuria gene accounting for the *SLC3A1* and *SLC7A9* mutation negative cases, it becomes obvious that other genetic factors should be responsible for the cystinuria phenotype in nearly 15% of patients.

## Background

Cystinuria (OMIM 220100) is a congenital disorder characterized by the defective renal reabsorption of cystine and other dibasic amino acids in the proximal renal tubule and in the epithelial cells of the gastrointestinal tract (for review: [1]). The resulting hyperexcretion of cystine leads to its precipitation in the distal tubule and formation of cystine stones. In adults cystine stones account for only 1–2% of all nephrolithiasis patients, whereas cystine stones account for 6–8% of pediatric urolithiasis patients [2].

So far, two autosomal genes have been identified to harbor genetic variants causing cystinuria. The *SLC3A1*/*rBAT* gene in 2p21 encodes the heavy subunit of the heterodimeric rBAT-b^0,+^AT transporter, the light chain is encoded by the *SLC7A9*/ *b*^*0,+*^*AT* gene in 19q12 [3–7]. The transporter is localized in the apical membrane of proximal tubules in the kidney and mediates the reabsorption of cystine, arginine, ornithine and lysine. Genomic variants in both genes have been identified in cystinuria patients. Whereas mutations in *SLC3A1* are commonly inherited in an autosomal recessive manner, *SLC7A9* alterations show a broad variability of inheritance, ranging from autosomal recessive to dominant. In some patients large genomic deletions in 2p21 affecting the *SLC3A1* gene and its neighbored *PREPL* gene can be detected, in that case urolithiasis is associated with hypotonia and further clinical symptoms (Hypotonia-Cystinuria syndrome - HCS, OMIM 606407).

Comprehensive mutation analyses in both genes allow the detection of genetic variants in more than 85% of patients, but a significant number of patients currently remain without a molecular diagnosis. In fact, the lack to detect pathogenic mutations can be explained by the applied methods and their different sensitivities, the influence of the ethnic origin on the distribution of mutations, and the autosomal dominant impact of some mutations in *SLC7A9* (and *SLC3A1*). However, they do not explain that a significant number of patients remains without any detectable mutation.

This observation as well as results of expression studies in the proximal renal tubule for rBAT and b^0,+^AT indicated that further subunits of the plasma membrane protein rBAT are involved in the renal cystine reabsorption. In particular, the opposing expression of rBAT and b^0,+^AT with a decline of rBAT expression from S3 towards the S1 segment and vice versa points to the existence of a further factor. With AGT1 as the second partner of rBAT in the S3 segment, the long-postulated partner of rBAT in the S3 segment of the renal proximal tubule cystine transport has recently been identified [8].

Consequently, *AGT1/ SLC7A13* has been suggested as a further candidate gene for cystinuria, mutations in this factor might explain the so far unsolved cases of cystinuria in which mutations in *SC3A1* and *SLC7A9* had been excluded. We here report on the results of our search for pathogenic genetic variants in *SLC7A13* in a cohort of 17 patients without disease-causing variations in the known cystinuria genes.

## Methods

In total, 103 patients from Germany, Turkey, Greece, Italy and Eastern Europe had been ascertained in precedent studies [9–15] aiming on the identification of *SLC3A1* and *SLC7A9* variants or in the course of routine molecular diagnostics of cystinuria because of (recurrent) nephrolithiasis or cystinuria. The study had been approved by the ethical committee of the Medical Faculty at the University Hospital Aachen (RWTH Aachen, EK302–16), Germany.

Analyses for disease-causing variants in *SLC3A1* and *SLC7A9* comprised Sanger sequencing analysis of their coding regions and the intron-exon boundaries (NM_000341.3, NM_014270.4), and search for whole exon deletions and duplications by multiplex ligation probe-dependent amplification (MLPA; kit P426-A1, mrc Holland, Amsterdam/NL).

The coding exons and the neighboring intron regions of *SCL7A13* (NM_138817.2) were analyzed by Sanger sequencing after PCR amplification. Primers are listed in Table 1, PCR as well as sequencing conditions are available on request.

**Table 1:** Primers used for Sanger sequencing of the coding sequences and exon-intron boundaries of the *SLC7A13* gene

Exon	Foward Primer	Reverse Primer	Length of PCR product
1	SLC7A13_1.1F: CTTTGCAGCTACATAGGCAGG SLC7A13_1.2F: TGGACATCCTTGTTTCTGGGG	SLC7A13_1.1R: TAGGCAGCTTTGGGACAGAG SLC7A13_1.2R: GGCTGGCATGATCTGATTCAG	471 537
2	SLC7A13_2F: TAAAATCATGCTTGTACCCC	SLC7A13_2R: AACAGTGGTTCTGACTGGTG	330
3	SLC7A13_3F: TCATTAGTATTTCTCTTTTAACAC	SLC7A13_3R: TGTGTTTCACAGTAACTGAG	541
4	SLC7A13_4F: TGCAGGTATCATTCATGGATGTTC	SLC7A13_4R: TGTTTAACCTTGATTTGGAATCTG	367

## Results

In 103 cystinuria patients, 83.5% had at least one *SLC3A1* or *SLC7A9* mutation (Table 2). In 49.5% (*n* = 51) of patients, two mutations could be observed, either as homozygosity / compound heterozygosity in one of the two genes or as mixed heterozygosity in both genes. In 17.5% (*n* = 18), one mutation was detected for which a penetrance in heterozygous state can be expected, i.e. the duplication of exons 5 to 9 in *SLC3A1* or *SLC7A9* mutations. In 16.5% (*n* = 17), only one mutation in *SLC3A1* was identified which would not explain the phenotype, in these cases a second mutation can be expected but is still undetected. In two patients we identified homozygosity / compound heterozygosity for large 2p21 deletions, these patients were molecularly diagnosed as HCS as both *SLC3A1* and *PREPL* were affected.

**Table 2:** Summary of the mutation detection results for *SLC3A1* and *SLC7A9* in a cohort of 103 cystinuria patients

	two mutations	one mutation explaining cystinuria^a^	only one mutation	no mutation
*SLC3A1*	39°	2	17	/
*SLC7A9*	11	16	/	/
mixed	1	/	/	/
total	51 (49.5%)	18 (17.5%)	17 (16.5%)	17 (16.5%)

^a^heterozygosity of the duplication of exons 5 to 9 in *SLC3A1* and of *SLC7A9* mutations without parallel occurrence of a second mutation has been reported to be sufficient to cause cystinuria; °including large HCS deletions

Mutation analysis for the two genes was negative in 16.5% of patients, and these 17 individuals (Table 3) were screened for pathogenic mutations in the coding sequences and the intron-exon boundaries of *SLC7A13*. However, with the exception of already known apathogenic polymorphisms (rs7814198, rs4419794, rs4621787, rs4546639, rs202114931, rs56993779, rs9656982) we did not detect any pathogenic genetic variant.

**Table 3:** Overview on the 17 patients screened for *SLC7A13* mutations

patient	ethnic origin	age at first stone	age at examination	number of recurrent stones	published in
Cys3	Italian	4 m	12 y	5–10	[9]
Cys4	Italian	1 y	5 y	< 5	[9]
Cys21	Italian	–	23 y	none	[9]
Cys23	Turkish	NA	30 y	< 5	[10]
Cys43	German	19 y	51 y	> 10	[10]
Cys58	Turkish	6 y	16 y	< 5	[14]
Cys63	German	13 m	3 y	5–10	[14]
Cys89	Russian	25 y	30 y	< 5	[14]
Cys98	German	3.5 y	5 y	< 5	[14]
Cys105	Russian	1.5 y	12 y	5–10	[14]
Cys116	Turkish	1.5 y	11 y	5–10	[12]
Cys128	Turkish	17 y	23 y	> 10	[12]
Cys152	German	NA	NA	NA	[12]
Cys161	Turkish	1 m	1 m	1	[12]
Cys181	Polish	3 y	9 y	> 10	[13]
Cys183	Polish	6 y	6 y	5–10	[13]
M20679	German	NA	52 y	1	–

Mutations in the *SLC3A1* and *SLC7A9* genes had been excluded before (see texts). The majority of patients was in included in precedent studies. (*NA* not assessed; *y* year, *m* months)

## Discussion

In contrast to the majority of kidney stones which occur sporadically, cystine stone formation and cystinuria have been suggested to be exclusively caused by genomic mutations. This assumption was supported by the identification of mutations in cystinuria patients in the genes coding the two subunits of the renal rBAT-b^0,+^AT transporter. However, with the exception of populations with founder mutations (e.g. [16]), the detection rate for mutations in the *SLC3A1* and *SLC7A9* genes never reaches 100%, and genetic variants in both genes account for 80–85% in the cystinuria population. This incomplete detection rate is certainly caused by methodological restrictions as non-coding regions (introns, promotor region) of the genes are commonly not investigated and the applied methods show different sensitivities. Furthermore, the penetrance of some mutations in both genes differs from the classical modes of Mendelian inheritance. In case of *SLC3A1*, mutations are generally autosomal recessively inherited, but at least the duplication of exons 5 to 9 in this gene has been suggested to function as an autosomal dominant allele [17]. For *SLC7A9* the situation is more complex, in this gene mutations can have both recessive as well as dominant effects. Thus, an autosomal dominant inheritance with incomplete penetrance has been suggested for variants in *SLC7A9*. In summary, the uncertainties to classify mutations in the already known cystinuria genes make the decision on the final detection rate difficult. Furthermore, it can be asked whether the detection of only one mutated allele in *SLC3A1* or *SLC7A9* is indeed sufficient to explain the cystinuria phenotype, or whether a second genomic variant in the same or another gene is required.

In addition to this unclear situation in patients with only one disease-causing mutation, there remains a considerable ratio of patients without a mutation in both genes. This observation provides strong evidence for the existence of a further factor mutations in which might cause cystinuria, but precedent mutation detection analyses in different candidate genes did not provide evidence for their involvement in the etiology of the disease (e.g. *SLC1A5*/ ATB(0), *SLC7A10*/ASC-1) [14, 18–21]. The recently described novel cystine transporter *SLC7A13*/AGT1 in the renal proximal tubule was a promising candidate to harbor pathogenic mutations in cystinuria patients. In particular, the observation that AGT1 serves as the second partner of the rBAT transporter in the S3 segment and its expression is consistent with the inversed expression of the so far known cystine transporter subunits *SLC3A1*/rBAT and *SLC7A9*/ b^0,+^AT [8] strongly points to *SLC7A13* as a candidate gene for cystinuria.

Despite these strong evidences, we could not confirm a relevant role of *SLC7A13* as a third gene contributing to the pathology of cystinuria. In our cohort which was comprehensively analysed for *SLC3A1* and *SLC7A9* variants, we could not detect any pathogenic mutation in the coding sequences and intron-exon boundaries of *SLC7A13*. In fact, we cannot exclude large deletions or duplications affecting whole exons, or variants in the non-coding regions of the gene. However, these types of mutations generally contribute only to a small proportion of the mutation spectrum of inherited diseases.

With the exclusion of *SLC7A13*/AGT1 as the third cystinuria gene, the question remains unanswered why analysis for the two cystinuria genes *SLC3A1* and *SLC7A9* succeeds only in up to 85%. In fact, this finding can be explained by ethnic differences in the distribution of mutations, and by methodological limitations. However, the comprehensive analysis for *SLC3A1* and *SLC7A9* mutations in a large number of studies (for review see: [1, 22]) does not indicate that a considerable number of genomic mutations in *SLC3A1* or *SLC7A9* has been missed. We therefore postulate that there is another genomic factor causing cystinuria. This factor might either been another so far unknown renal cystine transportert, or a genomic predisposition factor like a frequent polymorphism in one of the already known genes which – in combination with other predisposing elements (other genomic variants, life style) - causes the disease.

## Conclusion

With the exclusion of *SLC7A13*/AGT1 as the third cystinuria gene accounting for the *SLC3A1* and *SLC7A9* mutation negative cases, it becomes obvious that other genetic factors should be responsible for the cystinuria phenotype in nearly 15% of patients.
